# Muscular and Physical Response to an Agility and Repeated Sprint Tests According to the Level of Competition in Futsal Players

**DOI:** 10.3389/fpsyg.2020.583327

**Published:** 2020-12-18

**Authors:** Jorge García-Unanue, José Luis Felipe, David Bishop, Enrique Colino, Esther Ubago-Guisado, Jorge López-Fernández, Enrique Hernando, Leonor Gallardo, Javier Sánchez-Sánchez

**Affiliations:** ^1^IGOID Research Group, University of Castilla-La Mancha, Toledo, Spain; ^2^School of Sport Sciences, Universidad Europea de Madrid, Villaviciosa de Odón, Spain; ^3^Institute of Sport, Exercise and Active Living, Victoria University, Melbourne, VIC, Australia; ^4^School of Medical and Health Science, Edith Cowan University, Joondalup, WA, Australia; ^5^Centre for Sport, Exercise and Life Sciences, Coventry University, Coventry, United Kingdom

**Keywords:** repeated-sprint ability, tensiomyography, professional sport, sport performance, elite vs. amateur

## Abstract

The aim of this study was to evaluate the neuromuscular response to an agility and repeated sprint ability (RSA) test according to the level of competition in futsal players. A total of 33 players from two elite teams and one amateur team participated in the study. The participants completed an agility *t*-test, a 30 m-speed test, and a RSA test. A countermovement jump (CMJ) test and a tensiomyography test of the rectus femoris (RF) and biceps femoris (BF) of both legs were carried out before and after the tests. RSA test revealed better sprint times in elite players compared to amateurs in the seven bouts, as well as in the 30 m sprint and in the agility test (*p* < 0.05). Before the tests, elite players showed higher sustain time (Ts) in RF (+31.03 ms; ES: 0.76) and BF (+28.73 ms; ES: 0.73), higher half-relaxation time (Tr) in BF (+20.79 ms; ES: 0.94), and lower delay time (Td) in BF (−2 ms; ES: 1.19) compared to amateur players. However, post-test values did not present any significant differences (*p* > 0.05). In conclusion, elite players showed greater performance in the RSA test, in the 30 m tests and in the agility test compared to amateur players. The contractile properties were not a key factor in the RSA performance of the futsal players.

## Introduction

Futsal is a sport in which players have an average heart rate greater than 85% of their maximum (Rodrigues et al., 2011) and is characterized by a great number of high-intensity efforts such as sprint (actions run over 5.08 m/s), accelerations (actions with a speed increment over 2 m/s^2^) or decelerations (break actions run over 2 m/s^2^) (Caetano et al., 2015). A recent study using local positioning system revealed that Spanish top futsal players run between 12.30 and 17.54 m per min over 4.19 m/s and between 13.40 and 15.72 m per min with an acceleration over 1.12 m/s^2^ (Serrano et al., 2020). Moreover, these players also perform between 7.42 and 9.41 accelerations over 2 m/s^2^ per minute, execute between 6.94 and 9.12 decelerations over 2 m/s^2^ per minute or run between 0.58 and 0.88 sprints per minute; what might explain why VO_2max_ levels in futsal players can exceed 60 ml/kg/min (Castagna et al., 2009; Rodríguez-Ruiz et al., 2012).

These findings, as well as those from previous studies (Castagna et al., 2009; Castagna and Alvarez, 2010), show that players’ ability to maintain high-intensity actions in futsal matches is a key element of performance. In fact, futsal seems to demand a greater amount of high-intensity actions than other team sports like basketball, handball, or soccer (Naser et al., 2017). Thus, it can be expected high-intensity actions like sprints to be affected by a decrement in torque production of the knee flexor and the extensor muscles of futsal players due to acute fatigue (Dal Pupo et al., 2017). The unlimited changes allowed in futsal matches seems to counteract the effect of fatigue as variables like distance covered per minute, peak velocity, initial velocity, recovery time between sprints, sprints performed per minute, explosive distance per minute or number of accelerations, and decelerations per minute do not decrease in the second half of the game (Caetano et al., 2015; Serrano et al., 2020). However, the repeated sprints actions separated by a short period of rest (15, 30, 45, or 60 s) are frequent in futsal (Caetano et al., 2015), so players’ neuromuscular response and resistance to fatigue play a fundamental role in decisive actions of the game despite the unlimited changes (Loturco et al., 2015).

Repeated Sprint Ability (RSA) tests are considered as one of the main instrument to analyze the ability of futsal athletes to deal with repeated high-intense actions (Barbero-Alvarez et al., 2008; Sánchez-Sánchez et al., 2018). This test has proved to be useful to analyze the effect of resting between seasons in futsal players (Njororai, 2007) and to discriminate between playing level as those with lower performance decrease in the RSA test showed a higher number of high intensity actions during a real game situation (Carling et al., 2012). Existing evidence suggests that factors like explosive strength, muscle stiffness or intersegmental coordination of lower limbs might play a key role in the ability of athletes to cope with repeated sprints (Stojanovic et al., 2012; Dal Pupo et al., 2017) what might be affected by acute fatigue (Milioni et al., 2016). However, a recent study found that acute fatigue deteriorates neither muscle stiffness nor the intersegmental coordination of futsal players’ lower limbs despite it does damage some kinematics of lower limbs (Dal Pupo et al., 2017). Therefore, further research is needed to understand how futsal players’ lower limbs cope with the demands of repeated sprints.

The study of the neuromuscular responses of futsal players’ lower limbs to induced fatigue might be useful for this regard. The tensiomyography, a non-invasive technique that evaluates the muscle response to electrical stimuli (Sánchez-Sánchez et al., 2018) is useful to measure neuromuscular responses as it provides information on the rigidity of the muscle, contraction velocity, and state of fatigue (Miller et al., 2006; Rodrigues et al., 2011). The easiness of using this tool compared to others like electromyography or biomarkers combined with high levels of reproducibility and reliability for measuring vastus medialis, vastus lateralis, rectus femoris (RF), and biceps femoris (BF) (Warren et al., 2001; Spencer et al., 2005; Carrasco et al., 2011; Rodrigues et al., 2011), makes it a reliable method for comparing muscle response in different players (Sánchez-Sánchez et al., 2014; Wiewelhove et al., 2015).

Sánchez-Sánchez et al. (2018) reported that acute fatigue induced by a RSA test causes alterations in the contractile properties of the RF and BF of top futsal players. But it remains unknown if the alteration of lower limbs’ neuromuscular responses due to acute fatigue differ according to factors like the playing level or how contractile properties of lower limbs are related to the performance in RSA. For this reason, the aim of the present research was to evaluate the neuromuscular response to an agility and RSA test according to the level of competition in futsal players. It was hypothesized that elite players would obtain better results in the RSA test, as well as a lower decrease in performance during the test. Regarding the neuromuscular responses, it was hypothesized a significant relation of these responses with the RSA test results.

## Materials and Methods

### Participants

The sample was composed of three teams of the Spanish National Futsal League (LNFS), two elite teams and one amateur team. A total of 33 players were analyzed (stature: 175.48 ± 5.73 cm; body mass: 73.43 ± 5.93 kg; fat mass: 13.25 ± 3.57%). A stratified random sampling test was carried out according to the level of the teams (elite, *n* = 20; amateur, *n* = 13). Contact with the clubs was carried out through the LNFS. The ages of the participants ranged from 18 to 28 years (23.4 ± 4.42 years). Prior to participation in the study, all participants signed an informed consent form, which explained the test procedures and possible risks. The study protocol was approved by the Local Ethics Committee and was conducted in accordance with the Code of Ethics of the World Medical Association (Declaration of Helsinki).

### Experimental Design

The data collection process took place from September to December of 2019 after 1 month of pre-season training, and each team arranged with the researchers 3 days within this period to allow the players to perform the proposed test. The doctors of each team excluded all players who were in no condition to perform eccentric high-intensity exercises or had had an injury within the last 6 months. Previous to this study, players performed an initial pilot test to become familiar with the tests included in the study protocol. Participants agreed not to perform any exhausting activity 24-h before testing. Moreover, the head coach did not arrange any training drill 24-h before the testing. The tests were carried out on the futsal field.

### Experimental Protocol

Firstly, fat mass (g and %) and lean mass (g) of both legs were measured using bioelectrical impedance (Tanita BC418-MA, Tanita Corp., Tokyo, Japan). The SECA scale (model 711; SECA GmbH & Co., KG, Hamburg, Germany) was used to measure the height of the participants. Participants agreed to not to eat 3 h prior to the test, refrained from taking alcohol, or stimulant beverages for at least 15 h prior the test, drunk around 30 mL of water 90 min prior to the study in order to be hydrated when conducting the test and urinated a few minutes before the test (Poliszczuk et al., 2013; López-Fernández et al., 2020). Finally, composition for all subjects was estimated using the standard prediction equations rather than those designated for athletes.

#### Warm-Up Procedure

Participants performed a standard warm-up composed of 5 min of continuous running, 5 min of exercises of articulation mobility, and two 30-m sprints separated by 2 min of recovery. Stretching exercises were not part of the warm-up.

#### Baseline Assessments

Countermovement jumps (CMJ) were assessed using an infrared system (Optojump Next, Microgate, Bolzano, Italy). Participants placed both hands on their hips to avoid the influence of the movement of the arms on jump performance. Each player performed two jumps with 2 min recovery between jumps. The best of them was selected for statistical analysis.

Also, the muscular responses and the lateral symmetry of both the RF and BF were assessed by means of tensiomyography (TMG-100 System electrostimulator, TMG-BMC d.o.o., Ljubljana, Slovenia). This assessment provided the following information: the maximum radial displacement of the muscle belly (Dm), Contraction time (Tc), Delay time (Td), sustained contraction time (ts), and half-relaxation time (tr) of these muscles under basal conditions. Four stimuli of varying the amplitude (25, 50, 75 and 100 mAp) were given to both muscles for 1 ms. The rectus femoral was measured with the subject in a supine position, and with the knee maintained in a position of 120° flexion with the help of a foam triangular cushion. The biceps femoral was measured with the subject in the prone position, and with the knee maintained in a position of 5° flexion with the help of a foam cushion.

Dm (mm) is a parameter which reflects the maximum radial displacement of the muscle belly as a consequence of the muscle contraction and depends on the flexibility and the tone of the muscular tissue. Therefore, Dm values increase when the explosive force is developed, involving high movement amplitude, and they decrease under the conditions of a high muscular tone. Td (ms) is the time lapse between the transmission of the electric stimulus and the onset of muscle contraction (10% Dm). Tc (ms) is the time between the moment when the muscular contraction is 10% of the Dm and the moment when the contraction reaches 90% of the Dm. Ts (ms) is the time lapse when the muscle contraction remains above the 50% of the Dm. Finally, Tr (ms) is the time between the moment when the muscular contraction is 90% of the Dm and the moment when the contraction falls below 50% of the Dm. The electric stimulus is measured placing perpendicular to the muscle belly a digital Dc-Dc transducer Trans-Tek^®^ (GK 40, Panoptik d.o.o., Ljubliana, Slovenia), through two self-adhesive electrodes (TMG electrodes, TMG-BMC d.o.o. Ljubljana, Slovenia) placed equidistant at a distance of 50–60 mm from the digital transducer. Both positions of the sensor and the electrodes were marked with a permanent marker to ensure that all subsequent measurements were performed at the same point. All measurements were carried out by the same expert technician.

#### Performance Tests: The Agility *T*-Test and the 30-m Speed Test (RSA Test)

The Agility *t*-test was used to determine the performance in an action with changes of direction (Murayama et al., 2000). A pair of photocells (Witty, Microgate, Bolzano, Italy) was placed at the start line to measure total time.

Additionally, maximum speed was assessed using a 30-m speed test at the end of the warm-up. Total time was evaluated with two pairs of photocells located at 0 and 30 m. The RSA test included seven 30-m sprints with 20 s of recovery between sprints. Two pairs of photocells (Witty, Microgate, Bolzano, Italy) placed at 0 and 30 m were used. This test was performed according to the methodology proposed in previous studies (Barbero-Alvarez et al., 2008). The best sprint time (RSA_BEST_), the mean time (RSA_MEAN_), the total time (RSA_TT_), the percent sprint decrement {RSA_DEC_ = [(total sprint time – best time^∗^7)/best time^∗^7]^∗^100}, and the percent difference from best and worst sprint during the RSA test {RSA_CHANGE_ = [(worst time – best time)/best time]^∗^100} were also calculated (Chaouachi et al., 2010). The two previous sprints performed during the warm-up were used as control measure to guarantee players did the RSA test at maximum speed. If the time of the first sprint of the RSA test was higher (> 5%) than the best individual sprint performed prior to the beginning of the test, the RSA test was not considered valid and the player had to repeat the test after 5 min of recovery.

#### Post-performance Tests Assessment

Countermovement jumps (CMJ) and the tensiomyography were assesed again after the Agility *t*-test and the RSA test following the same protocol.

### Statistical Analysis

Data are presented as mean ± standard deviation. Statistical analyses were performed using SPSS (SPSS, version 21.0 for Windows, IBM Corp., New York, NY, United States). Normal distribution and homogeneity of variance was confirmed by Shapiro–Wilk test and Levene test, respectively. Differences in performance on the RSA test, the agility test, and the 30-m test between elite and amateur players were analyzed using a *t*-test for independent samples. Two-ways of variance (ANOVA) was used to analyze the differences in the tensiomyography and CMJ variables as a function of the moment (pre-post) and competitive level (elite-amateur). Bonferroni *post hoc* tests were used for the pairwise comparisons. In addition, confidence interval (CI of 95%) and the effect size (ES; Cohen’s d) were calculated to identify the magnitude of changes between groups. The ES was evaluated following the next criteria: 0–0.2 = trivial, 0.2–0.5 = small, 0.5–0.8 = moderate, and >0.8 significant (Cohen, 1992). Finally, the relationship between RSA scores and delta scores (post-pre in percentage) of tensiomyography variables was evaluated by linear regression analysis. One model was estimated for each tensiomyography variable, using the same independent variables in all of them (RSA_TT_, RSA_DEC_, and RSA_CHANGE_). The competitive level was included as categorical covariate (elite players were used as the reference group). The model did not present problems of heteroscedasticity. Moreover, variance inflation factor (VIF) was calculated to adjust the regression and prevent multicollinearity problems. The level of significance was established at *p* < 0.05.

## Results

**FIGURE 1:**
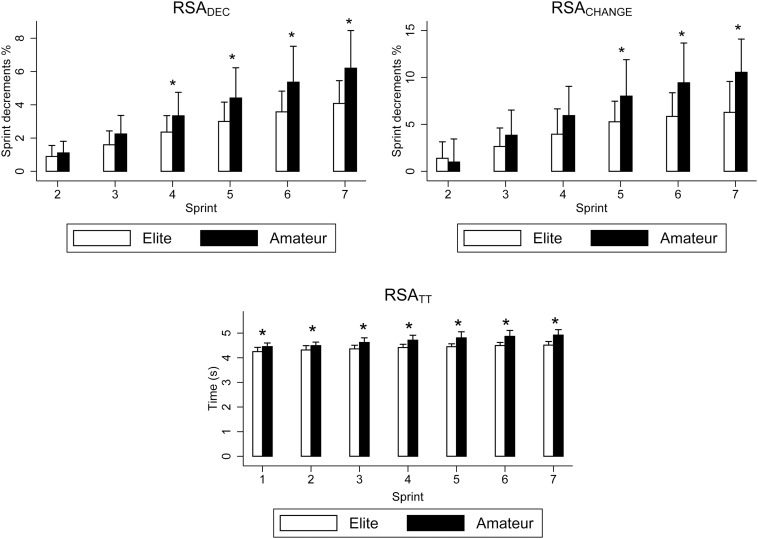
Differences in the results of the RSA test between elite (*n* = 20) and amateur (*n* = 13) futsal players. *p < 0.05.

The outcomes from the RSA test are displayed in Figure 1. Elite players showed lower RSA_TT_ (−2.95 s; ES: 1.59; CI 95%: −4.46 to −1.45; *p* < 0.001), RSA_BEST_ (−0.19 s; ES: 1.27; CI 95%: −0.30 to −0.08; *p* < 0.001) and RSA_MEAN_ (−0.30 s; ES: 1.89; CI 95%: −0.41 to −0.19; *p* < 0.001). Furthermore, they achieved better sprint times than amateur players from the first repetition (−0.06 s to −0.41 s; ES: 1.10–2.20; *p* < 0.01). On the other hand, amateur players showed higher RSA_DEC_ from the fourth sprint ahead (+0.97% to +2.12%; ES: 0.37–1.05; *p* < 0.05), and higher RSA_CHANGE_ from the fifth sprint ahead (+2.73 to +4.27; ES: 0.90–1.25; *p* < 0.05). Regarding the performance evaluation, the results showed better times in the 30 m sprint (−0.17 s; ES: 0.86; CI 95%: −0.31 to −0.03; *p* = 0.01) and agility test (−0.62 s; ES: 2.30; CI 95%: −0.81 to −0.42; *p* < 0.001) in elite players.

Table 1 shows the tensiomyography results before and after the RSA test according to the level of the players. The variance analysis revealed a significant reduction in the Td (−1.22 ms; ES: 0.87; CI 95%: 0.003–2.436; *p* = 0.049), Ts (−49.16 ms; ES: 1.21; CI 95%: 26.19–72.14; *p* < 0.001), and Tr (−9.85 ms; ES: 0.90; CI 95%: 10.97–48.73; *p* = 0.002) in the RF, and the Ts (−56.23 ms; ES: 1.31; CI 95%: 33.01–79.44; *p* < 0.001) in the BF, of elite players. On the other hand, amateur players showed a significant reduction of the Td (−1.71 ms; ES: 0.71; CI 95%: 0.20–3.22; *p* = 0.027) in the RF, and the Ts (−38.35 ms; ES: 2.10; CI 95%: 9.55–67.14; *p* = 0.010) in the BF after the RSA test. When comparing between groups, the basal test revealed elite players to have higher Ts in RF (+31.03 ms; ES: 0.76; CI 95%: 5.15–56.92; *p* = 0.020) and BF (+28.73 ms; ES: 0.73; CI 95%: 2.58–54.88; *p* = 0.032) and Tr (+20.79 ms; ES: 0.94; CI 95%: 1.60–39.98; *p* = 0.034) in BF. In addition, higher Td (+2.00 ms; ES: 1.19; CI 95%: 0.82–3.18; *p* = 0.001) in BF were found in amateur players in pre values. These differences disappeared after the RSA test (*p* > 0.05).

**TABLE 1:** Two-way ANOVA differences pre-post and elite-amateur.

		**Elite (*n* = 20)**		**Amateur (*n* = 13)**	
		**PRE**	**POST**	**PRE**	**POST**
RF	Td (ms)	23.68 ± 1.71	22.47 ± 1.06*	24.62 ± 3.14	22.91 ± 1.69*
	Tc (ms)	29.65 ± 6.38	26.80 ± 3.25	31.19 ± 6.48	28.60 ± 4.79
	Ts (ms)	122.90 ± 49.62#	73.73 ± 31.41*	91.87 ± 31.72	65.24 ± 18.94
	Tr (ms)	63.91 ± 37.48	34.07 ± 29.06*	46.92 ± 27.15	27.86 ± 17.61
	Dm (mm)	7.15 ± 2.53	8.08 ± 1.79	6.76 ± 2.06	7.32 ± 1.95
BF	Td (ms)	22.78 ± 1.09#	22.47 ± 1.26	24.78 ± 2.26	23.57 ± 2.16
	Tc (ms)	28.47 ± 7.92	29.03 ± 10.13	33.61 ± 7.75	32.23 ± 7.94
	Ts (ms)	209.34 ± 53.87#	153.11 ± 32.08*	180.61 ± 24.42	142.26 ± 12.18*
	Tr (ms)	62.70 ± 33.48#	48.97 ± 30.90	41.90 ± 10.73	45.30 ± 18.70
	Dm (mm)	5.70 ± 2.16	5.48 ± 2.03	5.95 ± 2.11	5.21 ± 1.72
CMJ (cm)		35.73 ± 5.97	34.00 ± 4.27	33.82 ± 4.20	31.77 ± 3.60

*Differences for pre-post for *p* < 0.05. #Differences between elite and amateur futsal players. RF, rectus femoris; BF, biceps femoris; Td, delay time; Tc, contraction time; Ts, sustain contraction time; Tr, half-relaxation time; Dm, maximum radial displacement of muscle; CMJ, countermovement jump.

The linear regression analysis comparing the relationship between RSA scores and delta scores (post-pre in percentage) of tensiomyography is displayed in Table 2. The RSA_TT_ and RSA_DEC_ showed a positive relation with Td of the RF (*p* < 0.05). Furthermore, RSA_TT_ evidenced a negative relation with the Ts of the BF (*p* < 0.05). However, the adjusted R2 is very low so it is the level of confidence.

**TABLE 2:** Regression analysis comparing the neuromuscular parameters and the outcomes from the repeated sprint ability (RSA) test standardized coefficients.

	**Td (%)**	**Tc (%)**	**Ts (%)**	**Tr (%)**	**Dm (%)**
**RF**					
Level	−1.12	−10.73	1.95	8.67	−21.82
RSA_TT_(s)	0.09	2.93*	−0.95	−5.35	−6.34
RSA_DEC_%	2.80	8.26*	5.03	6.65	6.46
RSA_CHANGE_%	−1.48	−3.59	0.08	1.53	0.50
Constant	−6.56	−89.78*	−28.62	76.67	216.04
Adjusted R2	0.10	0.35	0.17	0.14	0.08
**BF**					
Level	−1.82	−3.83	6.64	27.43	−7.04
RSA_TT_(s)	0.20	1.31	−2.03*	−1.88	−2.32
RSA_DEC_%	0.71	−0.50	4.25	10.11	−8.25
RSA_CHANGE_%	−0.90	−1.69	−1.09	−4.89	5.65
Constant	−1.53	−16.21	19.61	11.72	66.80
Adjusted R2	0.24	0.08	0.32	0.11	0.15

*p < 0.05. RF, rectus femoris; BF, biceps femoris; Td, delay time; Tc, contraction time; Ts, sustain contraction time; Tr, half-relaxation time; Dm, maximum radial displacement of muscle; Level: elite players were used as reference group.

## Discussion

The results of the present study show that elite futsal players have a higher performance in RSA test. Some differences in contractile properties of BF between elite and amateur players were found in the basal test. However, the regression analysis showed no clear relationship between RSA performance and tensiomyography variables as the few found associations displayed a low level of confidence.

The elite players obtained better results in the average time (RSA_MEAN_), total time (RSA_TT_), and best time sprint (RSA_BEST_) variables. In relation to performance deterioration, amateur players showed a greater effect of fatigue from the fourth sprint onward. This might be due to amateur players have lower levels of VO_2max_ and muscle glycogen concentration than elite layers (Balsom et al., 1999; Bishop and Spencer, 2004; Makaje et al., 2012).

1Previous investigations reported that Dm and Tc are the contractile properties that possess greater precision and sensibility when assessing the effects of training (Doğramaci et al., 2015; Wiewelhove et al., 2015). These studies coincide in that muscle fatigue is mainly characterized by increasing Dm and decreasing Tc. This relation would be caused by a loss in the efficiency in the excitation-contraction coupling, deterioration in membrane conduction properties, and a destruction of cellular properties (Doğramaci et al., 2015). This mechanism would cause a greater structural tension in the cell during resting state and an inability to completely active the contractile processes (Murayama et al., 2000; Byrne et al., 2004; Tous-Fajardo et al., 2010). Our result showed a slight increase of Dm and decrease of Tc in RF after completing the RSA test. However, the lack of significant differences between basal and post measures suggest that the RSA test conducted in this study did not produce a significant effect on the contractile properties of the studied muscles.

Regarding the Ts, our results show a general significant decrease after the RSA test in both amateur and elite players, existing also differences according to the level of competition of the players. In this sense, elite players showed a greater ability for maintaining muscle contraction (higher Ts) at basal state compared to amateur players in both the RF and the BF. Although these differences disappeared after completing the RSA, the Ts parameter of the BF might be susceptible of identifying differences in the players performance according to their competitive level. However, further studies are required due to the regression analysis did not show a strong relationship between the RSA outcomes and Ts of BF.

Shorter vales in Td has been suggested to indicate a higher ability to generate rapidly force in actions demanding repeated muscle contractions (Rey et al., 2012). This study is somehow in line with these assumptions as elite players displayed lower Td than amateur ones with significant differences for BF. However, the regression analysis does not support a the hypothesis of a previous study that suggested that higher Td might indicate a lower ability for repeated high-intention actions as those demanded by RSA test (Sánchez-Sánchez et al., 2018).

The sample of the present study was composed by amateur and elite players with similar basal contractile properties in the RF (no significant differences were found except in the Ts) but clearly different in the BF (Td, Ts, and Tr differ significantly). Therefore, it is logical to think that the differences in sprint performance observed in the present study between elite and amateur players could be partially caused by differences in the conditioning of the hamstring muscles, as these are the main muscles involved in controlling running activities and stabilizing the knee during turns and other actions (García-Manso et al., 2011). Moreover, in maximum sprint actions like those developed in this study, the hamstring muscles play a fundamental role in knee flexion as when running a maximum intensity, the heel elevation during the recovery phase is a rapid and explosive action (Girard et al., 2011). However, the regression analysis revealed none of the studied variables to have a strong association with the RSA performance what indicates that contractile properties of RF and BF cannot be used to this purpose.

To conclude, it is important to acknowledge the limited sample size of this study despite a stratified random sampling test was carried out according to the level of the teams. Additionally, the authors acknowledge that factors like the possible co-activation of neighboring muscles might affect the outcomes from the tensiomyography test (Martín-Rodríguez et al., 2017). However, to overcome this limitation the tests were conducted by the same technician who had a high experience using the tensiomyography.

## Practical Applications

In light of the results, we can conclude that elite players show greater performance in the RSA test, in the 30 m tests and in the agility test compared to amateur players. Also, elite players showed less performance decrement than amateur players from the fourth sprint onward in the RSA test. Regarding the neuromuscular profile between both populations, elite players showed differences in the contractile properties of BF in basal test. However, the regression analysis suggest that these differences do not play a key role in a RSA test in futsal players.

## Data Availability Statement

The raw data supporting the conclusions of this article will be made available by the authors, without undue reservation, to any qualified researcher.

## Ethics Statement

The studies involving human participants were reviewed and approved by European University of Madrid. The patients/participants provided their written informed consent to participate in this study.

## Author Contributions

JG-U, JS-S, and JL-F carried out the data acquisition process and drafted the manuscript. LG provided access to the measurement equipment and contributed to the design and work planning of the data acquisition process. JG-U, DB, JF, and EC performed the data analysis and interpretation of the results. JS-S, EH, and JL-F provided advice and critically reviewed the manuscript. LG and DB coordinated all parts, contributed to the data acquisition process and critically reviewed the manuscript. All authors have read and approved the content of the manuscript, contributed significantly to the research of the present manuscript, and approved the final submitted version of the manuscript.

## Conflict of Interest

The authors declare that the research was conducted in the absence of any commercial or financial relationships that could be construed as a potential conflict of interest.
